# A Novel Dual Expression Platform for High Throughput Functional Screening of Phage Libraries in Product like Format

**DOI:** 10.1371/journal.pone.0140691

**Published:** 2015-10-15

**Authors:** Xiaodong Xiao, Yan Chen, Sheila Mugabe, Changshou Gao, Christine Tkaczyk, Yariv Mazor, Peter Pavlik, Herren Wu, William Dall’Acqua, Partha Sarathi Chowdhury

**Affiliations:** 1 Dept. of Antibody Discovery and Protein Engineering, MedImmune, LLC., Gaithersburg, MD, 20878, United States of America; 2 Dept. of Biopharmaceutical Development, MedImmune, LLC., Gaithersburg, MD, 20878, United States of America; 3 Dept. of Infectious Diseases, MedImmune, LLC., Gaithersburg, MD, 20878, United States of America; The Scripps Research Institute and Sorrento Therapeutics, Inc., UNITED STATES

## Abstract

High throughput screenings of single chain Fv (scFv) antibody phage display libraries are currently done as soluble scFvs produced in *E*.*coli*. Due to endotoxin contaminations from bacterial cells these preparations cannot be reliably used in mammalian cell based assays. The monovalent nature and lack of Fc in soluble scFvs prevent functional assays that are dependent on target cross linking and/or Fc functions. A convenient approach is to convert scFvs into scFv.Fc fusion proteins and express them in mammalian cell lines for screening. This approach is low throughput and is only taken after primary screening of monovalent scFvs that are expressed in bacteria. There is no platform at present that combines the benefits of both bacterial and mammalian expression system for screening phage library output. We have, therefore, developed a novel dual expression vector, called pSplice, which can be used to express scFv.Fc fusion proteins both in *E*.*coli* and mammalian cell lines. The hallmark of the vector is an engineered intron which houses the bacterial promoter and signal peptide for expression and secretion of scFv.Fc in *E*.*coli*. When the vector is transfected into a mammalian cell line, the intron is efficiently spliced out resulting in a functional operon for expression and secretion of the scFv.Fc fusion protein into the culture medium. By applying basic knowledge of mammalian introns and splisosome, we designed this vector to enable screening of phage libraries in a product like format. Like IgG, the scFv.Fc fusion protein is bi-valent for the antigen and possesses Fc effector functions. Expression in *E*.*coli* maintains the speed of the bacterial expression platform and is used to triage clones based on binding and other assays that are not sensitive to endotoxin. Triaged clones are then expressed in a mammalian cell line without the need for any additional cloning steps. Conditioned media from the mammalian cell line containing the fusion proteins are then used for different types of cell based assays. Thus this system retains the speed of the current screening system for phage libraries and adds additional functionality to it.

## Introduction

The past two decades have experienced the development of a variety of antibody display platforms to enhance the antibody discovery process. These include phage display [1, 2] ribosomal display [3, 4], microbial display [5, 6] mammalian cell display [7–13], mRNA display [14], and DNA display [15]. The main reasons underlying the importance and interest in these libraries are as follows: (i) they are free of immune bias that is often encountered with immunization based hybridoma technology; (ii) they not only capture the natural immune repertoire but also exceeds it by virtue of combinatorial VH and VL pairings, (iii) they can be used to directly isolate monoclonal antibodies (MAb) almost against any molecular species including non-immunogenic and self-antigens and (iv) the specificity of the antibody can be largely tailored by introducing steps like de-selection and epitope bias, cross reactivity, affinity selection and the like in the selection/panning stage. The success of these antibody libraries is mainly dictated by their size. The large size in turn calls for the need to deep mine these libraries in a high throughput fashion. These opportunities and challenges are best balanced by phage antibody display library technology which can (i) attain very large size (~10^11^) compared to most other display platforms, (ii) enable selection at large density (10^12^_–_10^13^ phages/ml), (iii) is amenable to selection using purified biochemicals, whole cells, tissues and even live animals and (iv) can be screened at a very fast rate because of the rapidity of bacterial expression system. However, it has some limitations. These include (i) screening the antibodies as monomeric scFv or Fab fragments, (ii) difficulty using them in complex cell based biological assays due to endotoxin contamination from bacterial cells and (iii) inability to properly screen in biological assays that require bi valent binding or avidity and rely on Fc function.

Following a successful selection with a phage display library, conventional approaches have been to perform a high throughput primary screening as soluble Fab or scFv expressed in *E*.*coli* and then convert the potential hits into IgG for expression in mammalian system for more in-depth characterization and clone selection [16]. The drawbacks of this approach, as mentioned above, are the inherent limitation of screening as antibody fragments and the labor intensive process of converting antibody fragments into whole IgG [17]. As a result the full repertoire of the selected phage population cannot be tested [18]. All these contribute to significant attrition rate and reduced efficiency of the phage display platform. To overcome this limitation, the practice of screening phage library outputs as scFv.Fc fusion proteins is becoming a new trend [19–22] because these fusion proteins largely resemble IgGs in terms of valency, avidity and effector functions and enable tag free expression and detection. Because many of the functions of IgG or IgG like scFv.Fc molecules are dictated by post-translational modifications the scFv.Fc fusion proteins reported to date are expressed only in mammalian cells [19–22].

Phage library outputs typically range in size between 10^5^−10^6^ [18] clones with a significant proportion being non-specific to the target, also designated as background [23]. Therefore high throughput primary screening is very important for identifying antigen specific clones [24]. Because mammalian cell transfection and IgG expression is a slow process compared to bacterial expression platforms [25], screening phage library outputs as scFv.Fc fusion proteins made in mammalian cells compromises the speed and depth of screening library outputs. We therefore aimed to develop a vector that enables expression of scFv.Fc fusion proteins, both in *E*.*coli* and mammalian cell lines. The vector, called pSplice, caters to scFv display libraries, which is a popular format for phage libraries [26]. It includes two sets of control elements, each responsible for protein expression in bacterial and mammalian expression system. The salient feature of the vector is an intron with GT-AG as the splice donor and acceptor sites at its 5’ and 3’ end, respectively, for use by an U2 splicesomome [27]. In between these two sites, it contains a modified Lac promoter and pelB based signal peptide that guide the expression and secretion of scFv-Fc into culture medium of the bacteria in matters of hours. This allows for screening of thousands of clones in biochemical and cell based binding assays that are not sensitive to endotoxins. After triaging of the potential hits, the corresponding plasmids are isolated and directly used to transfect mammalian cell lines for protein expression without involving any additional cloning step. In the mammalian cell line the intron is spliced out from the pre-mRNA transcribed from the CMV promoter and a functional hybrid signal sequence is assembled and put in frame with the scFv.Fc gene. This then leads to constitutive expression and secretion of the fusion protein into the culture medium of mammalian cell line which can either be used directly or purified for more in depth functional assays such as growth inhibition, opsonophagocytosis (OPK), antibody dependent cell cytotoxicity (ADCC) and internalization studies. Thus by virtue of dual expression ability, the platform preserves the speed of the bacterial expression for primary screening of scFv.Fcs while enabling production of high quality and endotoxin free preparations of the same fusion proteins from mammalian cell line for more in depth secondary screening. It can also be used as a general tool to study the effect of bacterial and mammalian expression of any protein of interest.

## Materials and Methods

### Ethics statement

This study includes use of human serum. The serum was made from whole human blood. Blood from healthy volunteers was obtained with informed consent by venous puncture under MedImmune’s blood donation program. This was reviewed and approved by the institutional review board (ethics committee) before onset of the study. Human embryonic kidney cell line, HEK293F was obtained from Life Technologies (Grand Island, NY) and the human promyelocytic cell line HL-60 was obtained from the American Type Culture Collection (Manassas, VA).

### Vector construction

**Fig 1:**
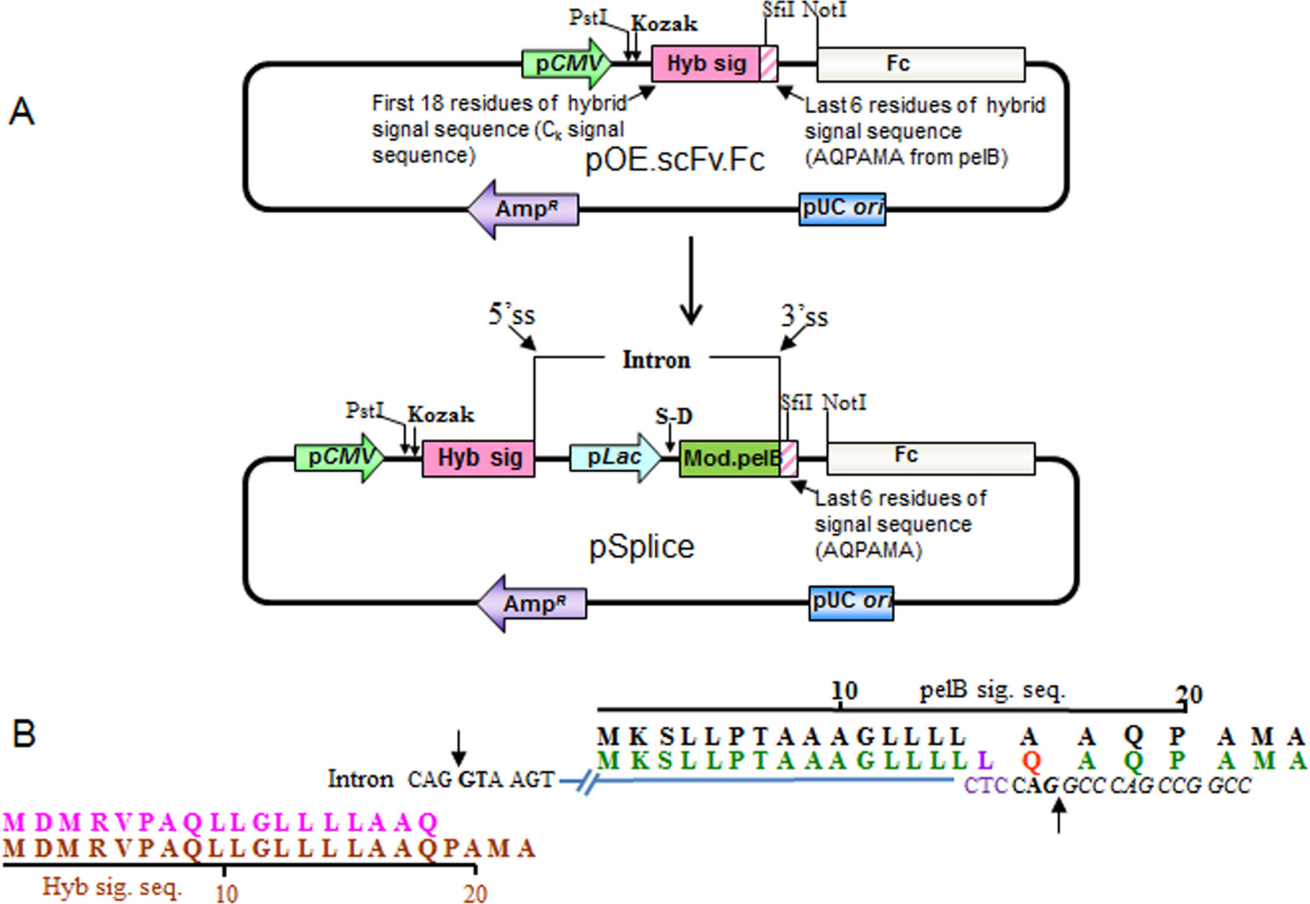
(A) Schematic representation of pSplice vector construction. pSplice is derived from pOE scFv.Fc vector. pOE scFv.Fc vector can express the fusion protein only in mammalian cells while pSplice vector can express the fusion protein both, in *E*. *coli* and mammalian cells. The hatched pink portion represents peptide sequence AQPAMA, which is common to the hybrid (Hyb sig) and the modified pelB (Mod pelB) signal sequences. Expression in the bacterial system is directed by the Lac promoter (light blue) and a modified pelB signal peptide (dark green followed by hatched pink), which are placed within an intron between the 5’ and 3’ splicing sites (ss). Production of scFv-Fc in 293F cells is controlled by CMV promoter (light green) and a hybrid signal peptide (solid pink followed by hatched pink) when the intron is spliced out in 293F cells. (B) Schematic representation of the intron and the main changes introduced in the signal sequences. In brown is the hybrid signal sequence of pOEscFv.Fc. In pink is the point where the open reading frame of hybrid signal sequence is disrupted by insertion of the intron (blue line). Bold letters (nucleotide sequence) and arrows in the intron represent the splice donor and acceptor sites. In black is the pelB signal sequence. Immediately below, in green is the modified pelB signal sequence. Within this peptide sequence an A to Q mutation is indicated in red. The corresponding codon creates the splice acceptor site. In magenta is an insertion of a leucine in pelB signal sequence. The corresponding codon creates a polypyrimidine site in the intron.

The pSplice vectors were constructed based on an in-house mammalian scFv-Fc expression vector, pOEscFv.Fc (unpublished). The expression and secretion of scFv.Fc from this vector is driven by a CMV promoter and a hybrid signal peptide sequence similar in design to that described by Yoon [20]. The first 18 amino acids of this signal peptide are from a human kappa light chain signal sequence and the last 6 amino acids are from the pelB signal sequence. The hybrid signal peptide coding sequence enables direct sub-cloning of scFv cassette as SfiI-NotI fragment from the phage display vector [28] to the pOEscFv.Fc expression vector. It can be used to express the scFv.Fc fusion protein only in mammalian cell lines such as HEK293F and CHO, much like what has been described by others [19–22]. To develop the pSplice vectors, a 394 base pair fragment of synthesized DNA was cloned between the PstI and SfiI sites of the parental pOEscFv.Fc plasmid (Fig 1A). This resulted in the introduction of an intron like sequence after the 18^th^ codon of the hybrid signal sequence. Within the intron is the Lac promoter, Shine-Dalgarno (S-D) and modified pelB signal sequences (Fig 1B). Based on knowledge about the major U2 splisosome machinery [27, 29–36] mutations were introduced in the lac promoter and the pelB signal sequence to augment the processing of the intron during expression in mammalian cell lines. We used the Splice site prediction tools of the Berkeley Drosophila Genome Project, http://www.fruitfly.org/seq_tools/splice.html and the Signal peptide prediction tools of CBS Prediction Servers, http://www.cbs.dtu.dk/services/SignalP/ to help us to predict the effects of the mutations that we designed. We focused on the following 4 components of U2 introns and built a series of iterative vectors to find one that worked the best:

1. The splice acceptor and donor sites (pSplice v.2)
2. The branch point (pSplice v.3)
3. The polypyrimidine or PPT tract (pSplice v.4) and
4. The G triplets and tetraplets or G-islands (pSplice v.5)

**Fig 2:**
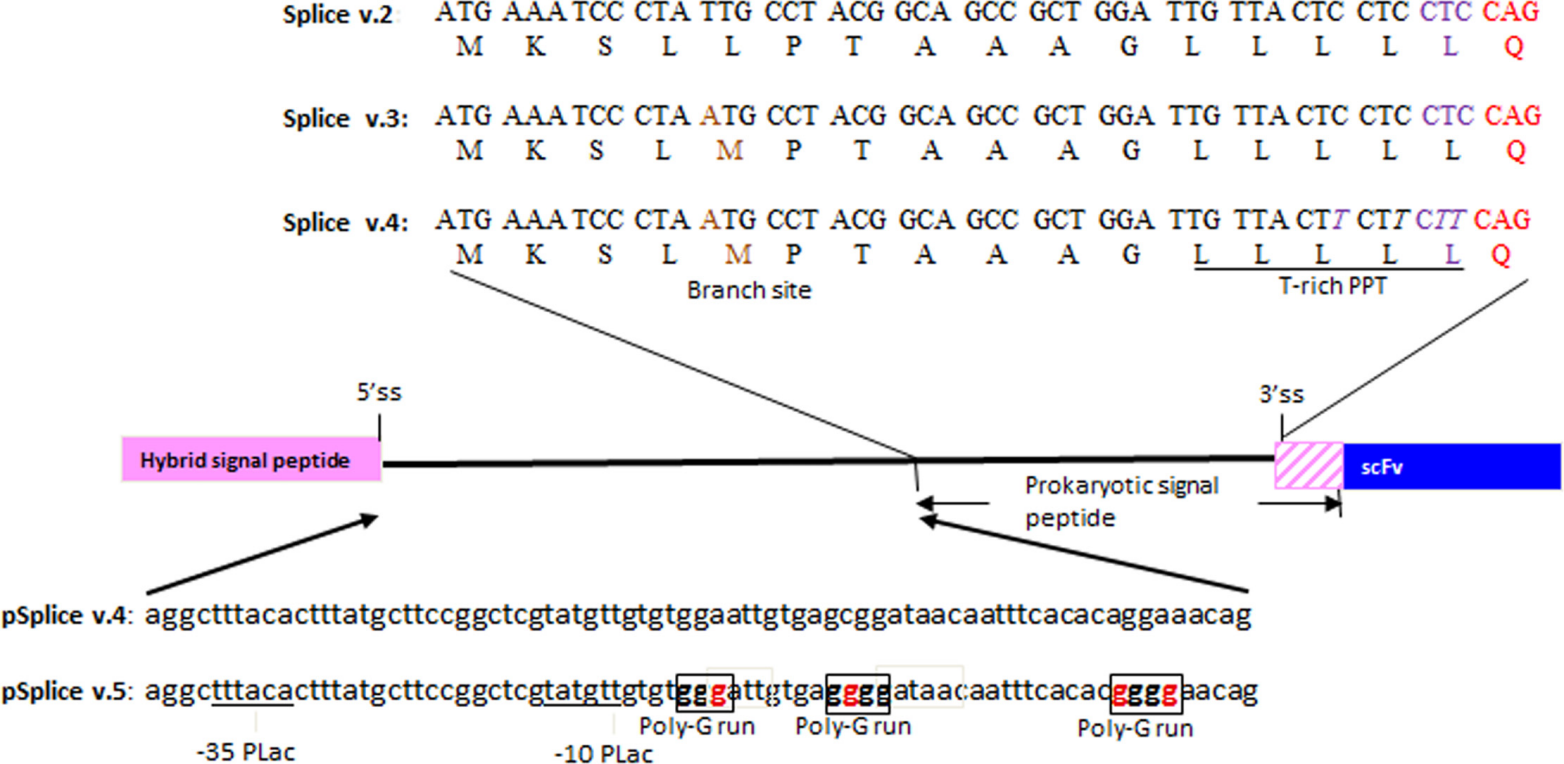
Upper panel: Codons and corresponding amino acids of pelB signal sequence showing splicing signal modifications in different versions of pSplice vectors. Introduction of branch point is indicated in brown. Introduction of the T-rich polypyrimidine tract (PPT) is shown in purple italic letters. Lower panel: Regions of engineering poly-G runs around -10 nucleotide of lac promoter are indicated by red letters in boxes.

All mutations were introduced by splicing-by-overlap extension PCRs using mutagenic primers. Two model scFv clones, clone 1 and 2 were selected for protein expression and characterization studies. They were cloned into the various pSplice vectors for evaluation (Fig 2). Details of the four pSplice vectors are described below.

1. pSplice v.2- This is shown in Fig 1B. The intron insertion points contain the splice donor and acceptor consensus sequences. The consensus splice donor site, A/CAGGTA/GAGT is created at the junction of the 18^th^ codon of the hybrid signal sequence and the start of the intron. To create the consensus splice acceptor site (a 15–18 nucleotide long pyrimidine rich tract followed by NCAGG, where N can be any nucleotide) the alanine at position 17 of the pelB sequence was mutated to a glutamine (indicated in red in Figs 1B and 2). In addition, a leucine was inserted upstream of it (indicated in magenta in Figs 1B and 2) to create a polypyrimidine tract (PPT) for the U2 splisosome. As a result of this design, the engineered pelB signal sequence was elongated by 1 amino acid (23 residues long) and the hybrid signal sequence was elongated by two amino acids (24 residues long).
2. pSplice v.3- This version differed from pSplice v.2 in that the 4^th^ position of the pelB signal sequence was mutated from a leucine to a methionine (indicated in brown in Fig 2). This was done to create a branch point for the action of the U2 splisosome [35].
3. pSplice v.4- This version differed from the pSplice v.3 in that the Cs at the wobble position of leucine codons 16–18 of the modified (23 residue long) pelB signal sequence were mutated to Ts to create a stronger PPT that promotes the use of adjacent 3’ splice sites (italics in Fig 2) [33].
4. pSplice v.5- This version differed from pSplice v.4 in that G-islands were created immediately downstream of the -10 position in the lac promoter (boxed in Fig 2). This version was selected for the highest expression of scFv.Fc fusion proteins out of a library of 8 different variants which differed in the location of the G-islands downstream of the splice donor site. The details of the other 7 variants are shown in S1 Fig. Multiple G-islands were inserted because they are known to have additive effects [30] in enhancing pre-mRNA splicing.

### scFv.Fc conversion

For validation of the platform, we used two scFv clones, number 1 and 2, which are derived from an in-house phage library [28] and specifically bind to a surface antigen on *Staphylococcus aureus*. We know from previous experience that clone 1 and 2 have high and low levels of expression, respectively, in the mammalian cell line, HEK293F. pSplice vectors 2, 3, 4, and 5 and phagemid DNA of clones 1 and 2 were digested first with NotI and then with SfiI following manufacturer’s instructions (NEB). Digested DNA including vector backbones from pSplice and inserts from phagemid were separated on a 1.2% agarose gel, recovered and cleaned with the gel purification kit (QIAGEN, Germantown, MD). The prepared vector and insert fragments were ligated using rapid DNA ligation kit (Roche Applied Science, Mannheim, Germany) at ambient temperature for one hour and the ligation reaction was transformed into BL21(λDE3) chemically competent cells (Invitrogen, Carlsbad, CA) following the standard transformation procedure. The transformed bacteria were plated to 2YT agar plates with 100 μg/ml carbenicillin and 2% glucose and incubated overnight at 37°C. Single colonies were first screened by PCR for the presence of the 750 bp scFv insert and sequence confirmed by DNA sequencing. For use of the platform in antibody lead isolation projects scFv genes from phage library output were cloned directly into pSplice v.5 and used to transform one shot TOP10 chemically competent cells ((Invitrogen, Carlsbad, CA)

### Expression of scFv.Fc fusion proteins from pSplice vectors

Expression of the fusion proteins from the four different pSplice vectors was studied in BL21(λDE3) *E*.*coli* strain and HEK293F mammalian cell line (Life Technologies, Grand Island, NY). For bacterial expression, *E*. *coli* colonies containing clone 1 and 2 scFv.Fc in the 4 different pSplice vectors were cultured in 100 μl of LB supplemented with 100 μg/ml carbenicillin and 2% glucose in 96 well round bottom plate (Becton Dickinson, Franklin Lake, NJ). After incubation overnight at 30°C with shaking at 500 rpm, 20 μl of the culture was transferred into 500 μl of MagicMedia *E*.*coli* Expression Medium (Invitrogen, Carlsbad, CA) supplemented with 100 μg/ml carbenicillin in deep well, 96 well plate and further incubated at 25°C with shaking at 850 rpm in a Multitron for 24 hours. Then 1/10 volume of PopCulture Reagent (Novagen, Darmstadt, Germany) supplemented with 1:1000 dilution of DNaseI (200 U/μl, Invitrogen, Carlsbad, CA) was added to each well and the shaking was continued for an additional 30 minutes. The plates were then centrifuged at 4500 rpm for 30 minutes before collecting supernatant for different assays.

For mammalian expression, HEK293F cell line was used. The same miniprep DNA that was used for bacterial transformation were used to transfect HEK293F cells using 293fectin Reagent (Invitrogen, Carlsbad, CA) according to the manufacturer’s instructions. Briefly, 1.5 μg of DNA was pre-incubated with 2 μl of 293fectin in 100 μl Opti-MEM (Invitrogen) for 20 minutes at ambient temperature and then added to 2 ml of 293F cells (at 10^6^ cells/ml) in 24-well round bottom polypropylene plates (GE healthcare, Piscataway, NJ). The plates were incubated at 37°C, 5% CO_2_ with shaking at 300 rpm for 3 days. For large scale scFv-Fc production, the amount of plasmid DNA, transfection reagent and HEK293F cells were scaled up proportionally and the transfected cells were cultured in flasks at 37°C, 5% CO2 with shaking at 120 rpm. The cell culture supernatant was collected at day 3 and day 6 after transfection. The scFv.Fc production was quantitated by Octet (ForteBio, Menlo Park, CA) using a Protein A Biosensor and a purified preparation of scFv.Fc as standard.

### scFv.Fc quantitation in HEK293F supernatant

The scFv.Fc production from HEK293F was determined by Octet (ForteBio, Menlo Park, CA) using basic quantitation method following the manufacture’s instruction. Briefly, Protein A biosensors (ForteBio, Menlo Park, CA) were pre-wet in 200 μl of Freestyle 293 media (Invitrogen, Carlsbad, CA) in a 96-well black polypropylene micro plate (Greiner bio-one, Monroe, NC) for 15 minutes. In corresponding wells of a matching plate, either known concentrations of scFv.Fc (as standard) in 200 μl of Freestyle 293F media or 200 μl of conditioned medium from transfected HEK293F cells (containing unknown concentrations of scFv.Fc) were added. After 15 minutes of equilibration, the probes were moved to scFv.Fc sample wells and binding was allowed to proceed for 2 minutes.

The samples were run using the pre-setting of Protein A standard assay. Once the run was completed, the standard curve was loaded and the scFv.Fc concentration was calculated by the data analysis software. Octet based quantification could not be done for the bacterial system because the bacterial culture supernatant was found to be incompatible with the Octet platform.

### SDS-PAGE analysis of scFv.Fc proteins expressed from bacterial and mammalian cells

scFv.Fc samples were prepared from BL21(λDE3) and HEK293F expression culture supernatant. 60 μl of Protein A Sepharose beads (GE Healthcare, Pittsburgh, PA) were added to 1 ml of BL21(λDE3) and HEK293F cell culture supernatant and incubated at room temperature for 30 minutes. After washing the beads 3 times with PBS, 60 μl of IgG Elution Buffer (Thermo Scientific, Rockford, IL) was added and incubated for 2 minutes. The beads were pelleted by centrifugation at 5000 rpm for 2 minutes in a microcentrifuge. About 60 μl of supernatant were collected and neutralized with 15 μl of 1M TrisHCL, pH8.0 (Invitrogen, Carlsbad, CA). 20 μl of the above prepared samples with and without Sample Reducing Agent (Invitrogen, Carlsbad, CA) were run on the NuPAGE 4–12% Bis-Tris gels in 1X MES SDS Running buffer (Novex by Life Technologies, Carlsbad, CA). After the run was complete, the gels were stained with SimplyBlue SafeStain (Invitrogen, Carlsbad, CA) to visualize the protein bands.

### ELISA for antigen binding

ELISA plates (Costar 3690, Corning, NY) were coated over night with NeutrAvidin biotin binding protein (Pierce, Rockford, IL) at 5 μg/ml in PBS, at 4°C. The plates were then blocked with 3% BSA in PBST (PBS with 0.1% Tween 20) for one hour at ambient temperature. Biotin labeled antigens (0.3 μg/ml) or negative control proteins were then added to the plate and captured for 1 hour followed by washing with PBST. scFv.Fc samples were then added to the plates and their binding was detected with HRP conjugated goat anti-human Fc secondary antibodies.

### Opsonophagocytosis (OPK) assay

HL-60 cells (ATCC, Manassas, VA) were differentiated in 3.5% dimethylformamide for 5 days in RPMI supplemented with 10% fetal bovine serum (InVitrogen) and containing no phenol red [37]. Clinical isolates of *S*. *aureus* were grown overnight in tryptic soy broth from a single colony, washed in cold PBS, and diluted 1:100 to an optical density_600 nm_ of 0.1. 10 μl of this S. aureus culture was incubated on ice for 30 minutes with 10 μl of the test and control scFv.Fc samples in 60 μl of Dulbecco’s Modified Eagle Medium (DMEM, InVitrogen) containing 0.1% gelatin (Sigma) in a U-bottom 96 well assay plate (Nunc). Human serum from healthy volunteer, that was pre-treated with *S*. *aureus* capsular polysaccharides from Type 5 Reynolds and Type 8 Wright (ATCC, Manassas, VA) isolates to clear any pre-existing anti *S*. *aureus* antibodies was used as the source of complement. 10 μl of this serum was added, along with 10 μl of HL-60 cells (10^7^/ml in HBSS, 0.1% gelatin) to each well of the assay plate at a final dilution of 1:100. Serum control wells included all components of the reaction except the scFv.Fc protein. Contents of the wells were mixed with a multi-channel pipet. At time 0 minute 10 μl from each well of the assay plate was serially diluted in PBS and plated on tryptic soy agar (TSA) plates (VWR International). The assay plate was incubated for an additional 60 minutes at 37°C with shaking at 100 rpm. At the end of 60 minute, 10 μl sample from each well of the assay plate was serially diluted in PBS for plating on TSA plate. Bacterial colonies were counted after incubation for 16 hours at 37°C. % of opsonophagocytic killing was calculated as follows:

$$100\;*\;\left(100-\left[{\text{cfu}}_{\text{time}\;60\text{min}}\right]/\left[{\text{cfu}}_{\text{time}\;0}\right]\right)$$

### N-terminal sequencing of scFv.Fc fusion proteins

Purified scFv-Fc proteins from mammalian host were subjected to protein sequencing by Edman degradation on a Model 494 cLC Precise protein/peptide sequencer with a UV-Visible detector. Samples were de-blocked with Pfu pyroglutamate aminopeptidase, 0.1% polysorbate 20, and 5X digestion buffer at 75°C for 4 hours and cooled. The de-blocked sample was washed and loaded onto the sequencer and sequenced for 15 residue-cycles. A standard mixture of 19 PTH-amino acids was injected onto the column for separation and this chromatogram was used as a standard for the PTH-AA peaks in each residue-cycle. The called sequence in each residue-cycle was based on the changes in the PTH-AA peak heights relative to the preceding and/or subsequent residue cycle.

### N-terminal sequencing by peptide mapping and LC-MS

Peptide mapping was used to determine N-terminal sequence of clone 1 and 2 scFv.Fc expressed in BL21(λDE3) and HEK293F cell line. Samples were denatured with 6M Guanidine HCl, 100mM Tris, pH 7.6 and reduced with DTT at 37°C for 30 minutes. The reduced cysteines were alkylated with iodoactemide at room temperature for 30 minutes, desalted and buffer exchanged to a 2M Urea/ Tris buffer, pH 7.6. The buffer exchanged samples were digested with trypsin to an enzyme: substrate ratio of 1:20 for 4 hours at 37°C. After digestion, 4% of TFA was added to quench the reaction.

A Waters Acquity UPLC coupled to a Thermo LTQ-Orbitrap mass spectrometer was used for LC-MS analysis. The mobile phase A was 0.02% TFA in water and mobile phase B was 0.02% TFA in acetonitrile. The digested peptides were separated on an Acquity C18, 2.1x150mm column at a flow rate of 0.2ml/min at 55°C and monitored using a UV detector and electrospray ionization mass spectrometer in positive ion mode.

The N-terminal signal peptide was identified by its mass, corresponding to the amino acid composition and the fragmentation masses determined by MS and MS/MS data. Data processing was done using Thermo Scientific’s software X-Calibur for manual processing and Proteome Discoverer 1.3 for automated search to confirm the fragments.

## Results

### pSplice vectors expressed and secreted scFv.Fc fusion proteins in *E*.*coli*

**Fig 3:**
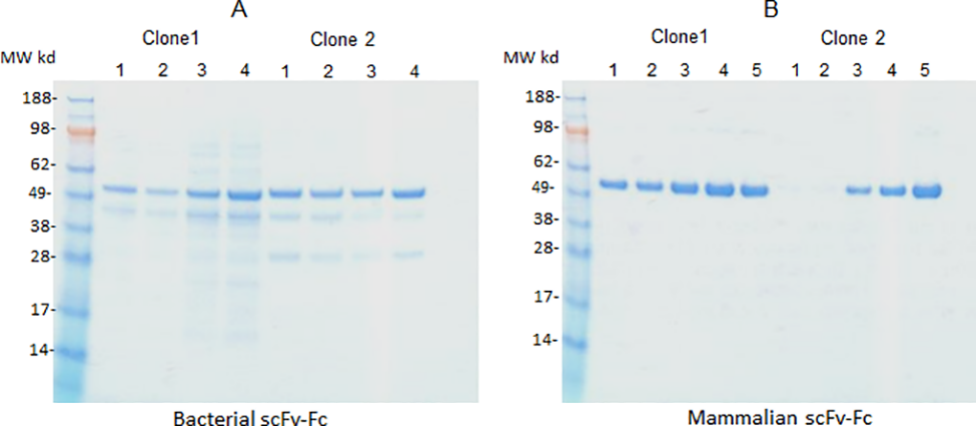
SDS-PAGE analysis of pSplice vectors in *E*.*coli* and 293F cells. (A) Non reducing SDS-PAGE of scFv.Fc of clone 1 and 2 expressed from pSplice version 2, 3, 4 and 5 vectors in *E*.*coli* (lanes 1, 2, 3 and 4, respectively). (B) Non reducing SDS-PAGE of scFv.Fc of clone 1 and 2 expressed from pSplice version 2, 3, 4 and 5 vectors and the parental pOEscFv.Fc (lanes 1, 2, 3, 4 and 5, respectively) in 293F cells. Mammalian expression vector, pOEscFv.Fc is used to compare how the dual expressing pSplice vectors perform in comparison to pOEscFv.Fc vector.

Because engineering of the intron changed the pelB signal sequence, we studied expression and secretion of 2 different scFv.Fc clones (clone 1 and 2) from the 4 different pSplice vectors (pSplice v.2, v.3, v.4 and v.5) in BL21(λDE3). As shown in Fig 3A the scFv.Fc fusion proteins were produced by all 4 pSplice vectors. For clone 1, but not for clone 2, pSplice v. 2 and v.3 produced slightly less protein than v.4 and v.5. pSplice v.3-v.5 code for the same signal peptide and differ from version 2 at position 4 of the pelB sequence (L4M). Since the expression of the fusion protein from the different vectors is about the same for clone 2, it appears that the modifications made in the pelB signal sequence (L4M, L16 insertion and A17Q) did not perturb its function.

We have observed that any scFv.Fc clone can be expressed in *E*.*coli*. In S2 Fig we present results of bacterial expression from pSplice v.4 and v.5 on a panel of 10 clones from an actual antibody discovery campaign against target X found on human cancer cells. This was done by antigen binding ELISA which is the assay of choice for primary screening.

### Intron engineering had marked effect on the expression of scFv.Fc fusion proteins in HEK293F cells

**Fig 4:**
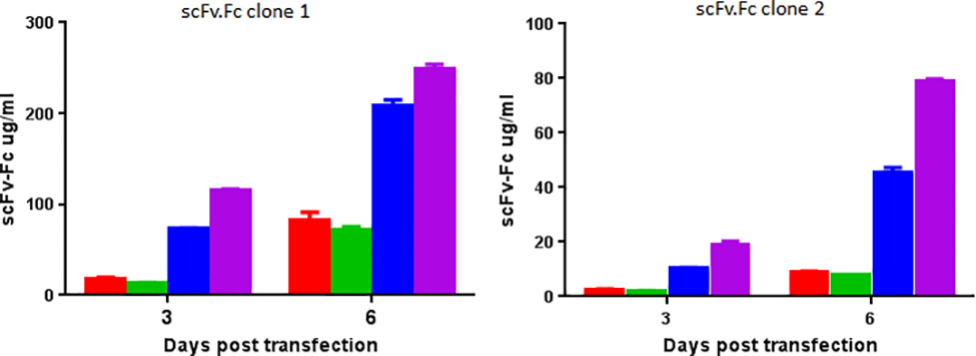
Expression levels of scFv.Fcs in mammalian cells. Octet based quantitation of expression level at day 3 and 6 post-transfection of scFv.Fc clone 1 and 2 from pSplice version 2 (red), 3 (green), 4 blue) and 5 (magenta) vectors. The mean values for each group are derived from experiments done in triplicates and the s.d. is represented by error bars.

Figs 3B and 4 and S3 Fig show the effects of intron engineering on the expression of scFv.Fc from clones 1 and 2 in HEK293F cells. For both the clones no difference was noted between pSplice v.2 and v.3. This indicated that the introduction of the intron branch point in pSplice v.3 did not influence protein expression significantly. In contrast, expression from pSplice v. 4 was considerably better than pSplice v.2 and v.3. pSplice v.5 gave the best expression level of all the vectors. We also compared expression from pSplice v.4 and v.5 on a panel of 10 clones from an actual antibody discovery campaign against target X found on human cancer cells. Expression from pSplice v.5 was higher for all 10 scFv.Fcs (S3 Fig). These results indicate that the introduction of a T-rich PPT and G-islands in the intron played a key role in influencing the expression level of scFv.Fc fusion protein. The expression level we achieved with the pSplicev.5 ranged from about 10 to over 150 μg/ml. These levels are more than sufficient for any *in vitro* functional assay.

### scFv.Fc fusion proteins expressed from *E*.*coli* and mammalian cells bound antigen

**Fig 5:**
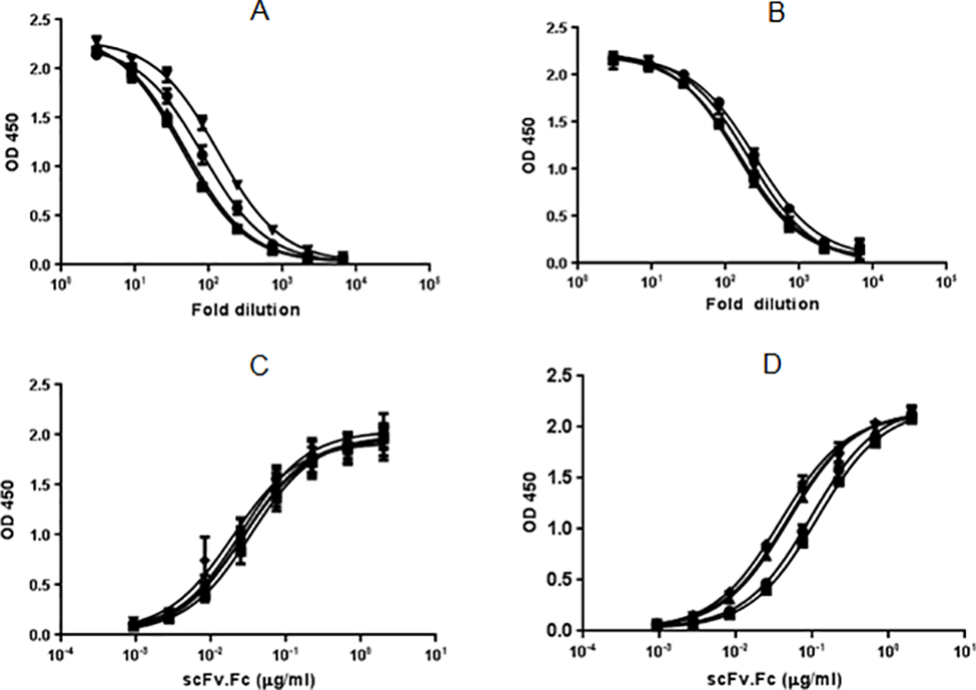
Antigen binding activity of scFv.Fc of clones 1 and 2 expressed from different pSplice vectors in bacteria (A and B, respectively) and in 293F cells (C and D, respectively). The symbols represent the following: circle-pSplice v.2; square- pSplice v.3; triangle- pSplice v.4; inverted triangle- pSplice v.5 and diamond- pOEscFv.Fc (only for mammalian expression in C and D). The scFv.Fc proteins did not bind to a negative control antigen (shown in S3 Fig). The mean values for each group are derived from experiments done in triplicates and the s.d. is represented by error bars.

Supernatants of BL21(λDE3) and HEK293F cells transformed/transfected with different pSplice vectors coding for the two scFv.Fc proteins were tested for antigen binding by ELISA. Because quantitation of the fusion protein in bacterial supernatants could not be done precisely, these samples were studied by serial dilution. As shown in Fig 5A and 5B and S2 Fig the supernatant of the bacterial culture contained enough scFv.Fc proteins that they could be diluted nearly 1000 fold to detect antigen binding activity. This is more than enough for high throughput primary screening for a variety of binding assays. Similarly supernatants from the HEK293F cells expressing the fusion proteins showed good antigen binding activity (Fig 5C and 5D). The antigen binding activity of the scFv.Fc fusion proteins from *E*.*coli* and HEK293F cells were specific since they did not bind to an irrelevant antigen (S4 Fig).

### scFv.Fc fusion proteins in culture supernatants of HEK293F cells showed opsonophagocytosis activity

**Fig 6:**
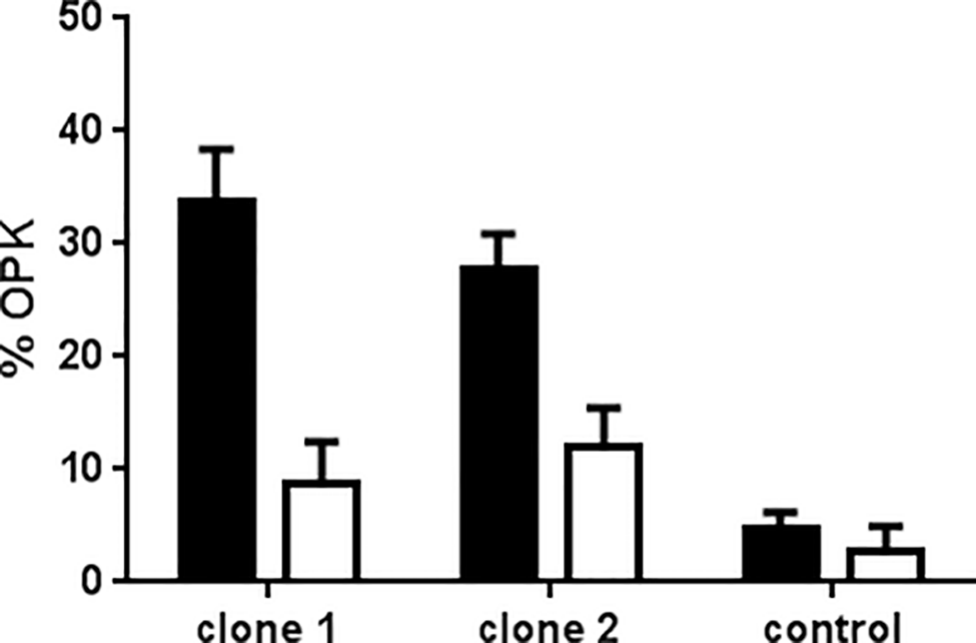
scFv.Fcs expressed in mammalian cells are biologically active. Opsonophagocytosis activity of scFv.Fc fusion proteins for clone 1, clone 2 and negative control clone in supernatants from 293F cells transfected with the respective pSplicev.5 plasmid. Black and white bars represent OPK activity of scFv.Fc at 10 μg/ml and 1 μg/ml, respectively. The mean values for each group are derived from experiments done in triplicates and the s.d. is represented by error bars.

Supernatants of HEK293F cells transfected with pSplice v.5 of clones 1 and 2 as well as a negative control scFv.Fc were tested for opsonophagocytosis (OPK) activity which is a function of the Fc segment. As shown in Fig 6, clones 1 and 2 but not a negative control scFv.Fc sample showed potent OPK activity. This indicates that pSplice vector expresses fusion proteins in HEK293F cells that can be tested for Fc based biological activities.

### scFv.Fc fusion proteins expressed in bacteria and mammalian cells had the correct N-terminus

**Fig 7:**
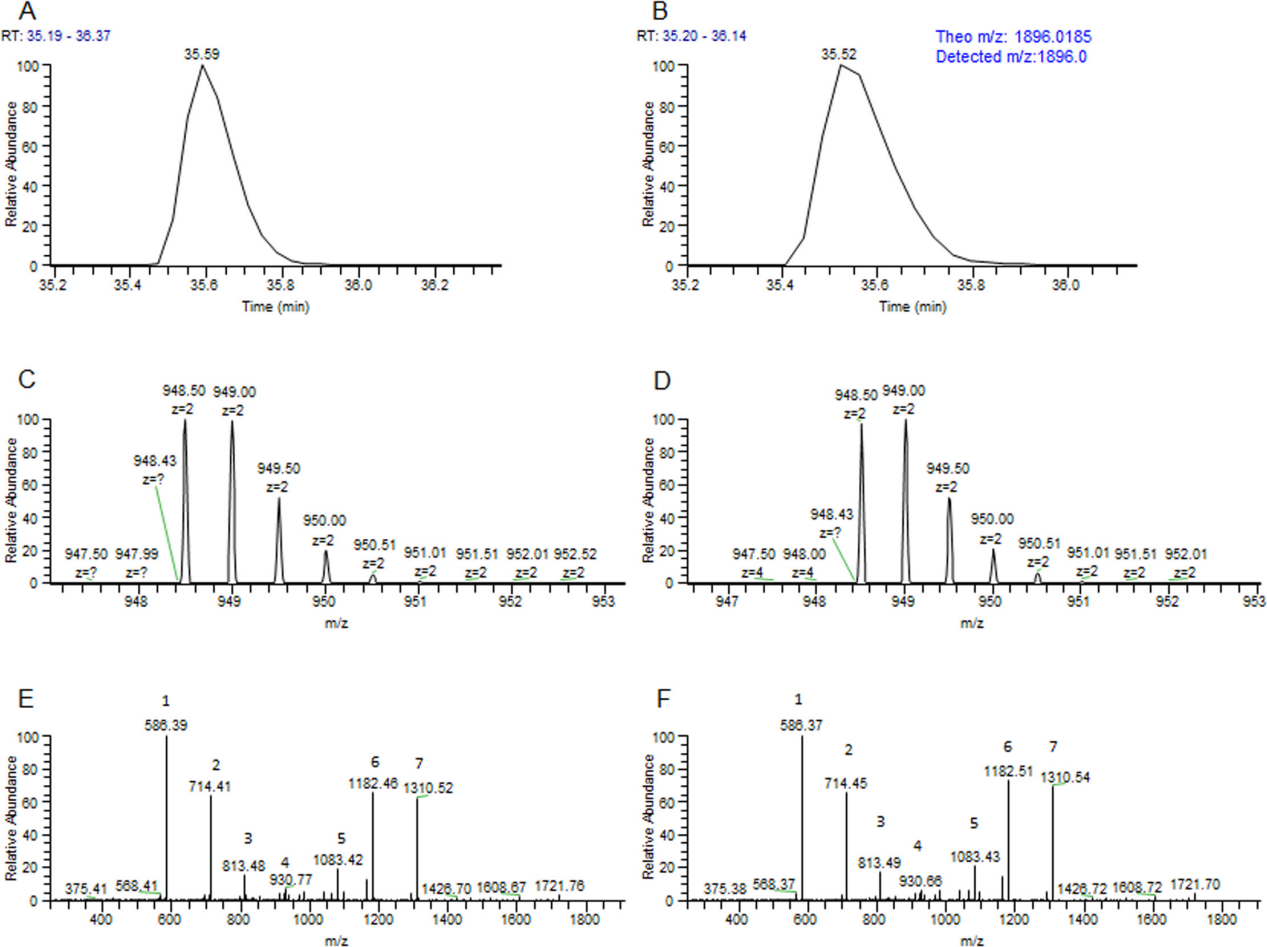
Upper panel: H1 peak (properly processed N-terminus corresponding to EVQLLESGGGLVQPGGSLR) after UPLC separation of tryptic peptides of clone 2 scFv.Fc expressed in bacteria (A) and 293F cells (B). Peak corresponding to the estimated molecular weight of a correctly matured N-terminus is shown. Middle panel: (C) and (D) MS spectra of (A) and (B) in the upper panel. The estimated molecular weight for the H1 peptide with a charge of +1 is expected to be 1896.0185 da. The m/z of the major peaks in (C) and (D) correspond closely to the peptides shown in (A) and (B), respectively. Lower panel: (E) and (F) ms/ms spectra of the same sample in (A) and (B). The different peptides that the labelled peaks correspond to are as follows: Peak 1: PGGSLR; peak 2: QPGGSLR; peak 3: VQPGGSLR; peak 4: EVQLLESG; peak 5: SGGGLVQPGGSLR; peak 6: LESGGGLVQPGGSLR and peak 7: LLESGGGLVQPGGSLR. Automated and manual search for improperly processed signal peptide yielded only about 0.4% of AMA-H1 peptide in scFv.Fc purified from 293F cells (not shown).

Mass spectrometry of peptide fragments from purified scFv.Fc fusion proteins of clones 1 and 2 expressed in BL21(λDE3) and HEK293F cells identified peptides corresponding to the processed N-terminus of the VH chain (the orientation of the antibody chains in our scFv library is VH-Linker-VL) (Fig 7). No peptides corresponding to part or whole of the signal peptide were identified for proteins expressed in BL21(λDE3). The same was true for proteins expressed in HEK293F cells except for a minute fraction (<1%) of clone 2 which had 3 amino acids from signal peptide attached to the N-terminus of VH (result not shown). For fusion proteins made in HEK293F cells we also did N-terminal sequencing for the first 15 amino acids. The result showed the correct N-terminus for the VH domains (result not shown). These results indicate that the modifications in the pelB and the hybrid signal sequence did not affect processing of the signal peptide in *E*.*coli* and mammalian cell, respectively.

## Discussion

Using basic knowledge of the U2 splisosome, we have successfully built a vector that enables for the first time high throughput functional screening of phage scFv library outputs as dimeric scFv.Fc proteins. We believe this will remove many of the artifacts of conventional monovalent scFv screenings. The development of the vector was challenging because expression and secretion of scFv.Fc proteins in two different hosts was achieved by engineering an intron that (i) contained the control elements for bacterial expression and secretion and (ii) could be spliced out properly in mammalian expression system, restoring a functional operon for expression and secretion of scFv.Fc proteins. This required us to engineer the intron in a way that does not affect the functional integrity of the lac promoter and the pelB signal sequence.

Mechanisms for intron removal by pre-mRNA splicing require precise intron-exon borders as well as splicing enhancer sequences within the intron [27, 29–35]. We have therefore explored 4 important components of the splicing machinery, namely, the splice donor and acceptor sites which define the borders of the intron and the PPT, the branch point and the G-islands which are thought to act as enhancers for intron splicing. In our studies we found that the T-rich PPT and the G-islands were most useful in improving expression while the branch point did not have any effect. This is indicated by the low level of expression seen from pSplice v.3 which has the branch point but substantial expression from pSplice v.4, which has the T-rich PPT and highest level of expression from pSplice v.5 which has the G-islands in addition to branch point and the T-rich PPT. This is in agreement with other reports which state that the G-islands and the T-rich PPT play more important role in inducing use of the closest splice donor and acceptor sites, respectively, compared to the branch point [30, 33, and 36]. However, because we did not make vectors that contain only the PPT or only G-islands we cannot definitively identify the relative contribution of each of these components in the context of the pSplice vectors.

In our efforts to engineer the intron for improving expression level, we aimed to minimize changes within the signal sequences and the lac promoter. For example, during creation of the PPT we chose to insert a leucine in the pelB signal sequence (position 16 in Fig 1B) instead of proline, serine and phenylalanine (all of which could create a pyrimidine rich codon and support RNA splicing) because insertion of the leucine was predicted to have no effect on the signal peptide processing, as opposed to the other 3 amino acids. Likewise we tried to engineer the branch point only on codon 4 of the pelB because this was the only position that could be mutated to conform to a branch point consensus without significantly affecting pelB signal peptide processing in *E*.*coli*. However, as mentioned before, introduction of the branch point did not lead to any improvement in the expression levels of the fusion proteins. We did not try other positions for branch point engineering because for mammalian expression the branch point does not have a big impact when the PPT is strong [36].

Engineering of the intron created substitution and insertion of amino acids in the pelB and hybrid signal sequences. Through mass spectrometry and N-terminal sequencing we showed that these changes did not affect the processing of the signal sequences in *E*.*coli* and HEK293F cells. This result indicates that the use of the splice site and signal peptide prediction tools for engineering the intron was important and justified.

The expression levels obtained by pSplice v.4 and v.5 in *E*.*coli* and HEK293F cells are sufficient to enable a variety of high through put assays. We have now used the pSplice v.4 and v.5 vectors in multiple lead isolation campaigns against growth factor receptors, enzymes and bacterial pathogenic factors. The main advantage of the platform is the speed, throughputness and breadth of assay capabilities that it offers compared to the traditional scFv screening. In work that we plan to publish separately, we have found the platform to be useful in discovering multiple hits by employing a variety of biochemical and cell based assays that are not possible with monovalent scFvs. For example, in one of the challenging projects against a complex growth factor receptor, we screened over 25000 bacterial colonies expressing scFv.Fc proteins, in 384-well plate format to identify human, cynomolgus and mouse cross reactive clones. A little over 3000 clones that were found to be cross reactive were screened by FACS for their ability to bind live cells. 77 clones that were FACS positive were then purified after expression in HEK293F cells for screening in a functional growth inhibition assay which led to the identification of 3 lead molecules. In a conventional periprep based scFv screening approach, this would have been a monumental task because 384-well plate format is not compatible with periprep generation for carrying out multiple binding studies. Because no sub-cloning for mammalian expression was needed the work could progress rapidly to identification of leads through FACS and cell based activity assay. We have also shown that for programs where Fc function, such as OPK, is desired, the supernatant from transfected HEK293F cells can be directly used to identify active hits. Because most therapeutic antibodies are formatted as IgG, our future plan is to evolve this dual expression system from screening phage library outputs in product-like scFv.Fc format to screening in product format as IgGs. This would entail batch reformatting of scFvs into IgGs and expressing them both in *E*.*coli* and mammalian cell lines from the same vector.

## Supporting Information

S1 Fig Sequences of 8 constructs that differ in the location of the G-islands immediately downstream of the splice donor site. pSpliceV5.1–1 gave the highest expression of the scFv.Fc fusion protein in 293F cells and was named pSplice v.5
(TIF)

S2 Fig Comparison of antigen binding activity of 10 different scFv.Fc fusion proteins (binding to target X) expressed from pSplice v.4 or v.5 vectors in E. coli, BL21(λDE3). Antigen binding activity of clones X1-X10 from pSplice v.4 (A) and pSplice v.5 (B) binding to their cognate antigen. Control sample, Clone 1 scFv.Fc, does not bind to this antigen. (C) and (D) Lack of binding activity of clones X1-X10 from pSplice v.4 and pSplice V.5, respectively, to a negative control protein (antigen for Clone 1). Note that clones X1-X10 do not bind to the antigen for Clone 1 but Clone 1 scFv.Fc show dose-dependent binding to its own antigen. The mean values for each group are derived from experiments done in triplicates and the s.d. is represented by error bars.
(TIF)

S3 Fig Comparison of 10 different scFv.Fc expression level in 293F cells transfected with pSplice v.4 and V.5 vectors. The mean values for each group are derived from experiments done in triplicates and the s.d. is represented by error bars.
(TIF)

S4 Fig scFv.Fc of clones 1 and 2 expressed from different pSplice vectors in bacteria (A and B, respectively) and in 293F cells (C and D, respectively) do not bind to a negative control antigen. The mean values for each group are derived from experiments done in triplicates and the s.d. is represented by error bars.
(TIF)
