# Supplementary figures and images for: A Novel Dual Expression Platform for High Throughput Functional Screening of Phage Libraries in Product like Format
Source: PLoS One. 2015 Oct 15;10(10):e0140691. doi: 10.1371/journal.pone.0140691 (PMC4607404; doi:10.1371/journal.pone.0140691)

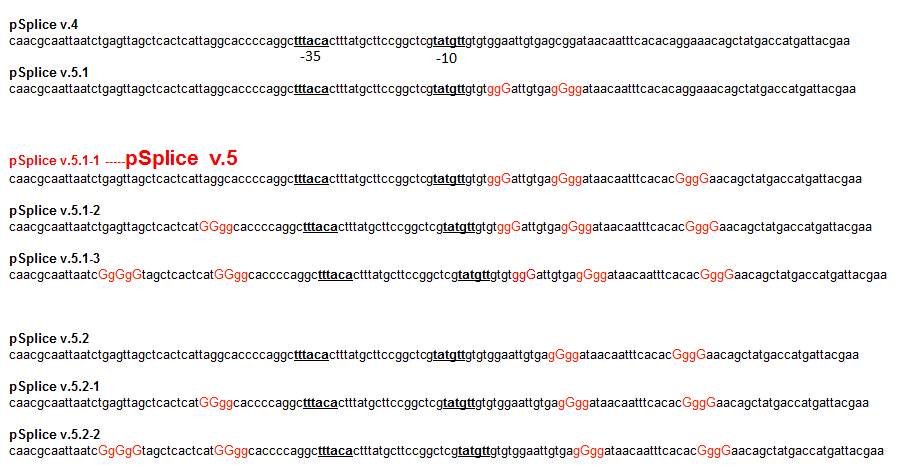

Supplement: S1 Fig — pSpliceV5.1–1 gave the highest expression of the scFv.Fc fusion protein in 293F cells and was named pSplice v.5 (TIF) [file pone.0140691.s001.tif]

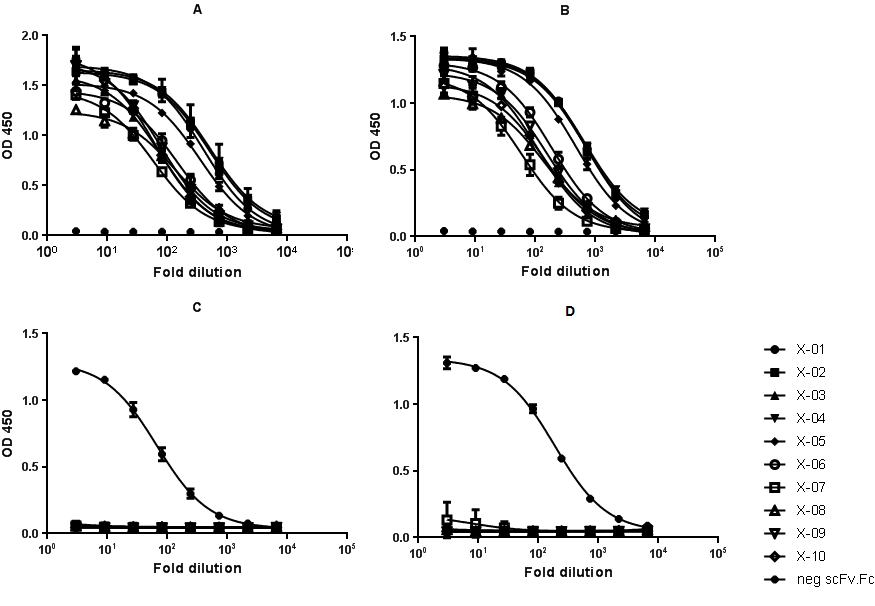

Supplement: S2 Fig — Antigen binding activity of clones X1-X10 from pSplice v.4 (A) and pSplice v.5 (B) binding to their cognate antigen. Control sample, Clone 1 scFv.Fc, does not bind to this antigen. (C) and (D) Lack of binding activity of clones X1-X10 from pSplice v.4 and pSplice V.5, respectively, to a negative control protein (antigen for Clone 1). Note that clones X1-X10 do not bind to the antigen for Clone 1 but Clone 1 scFv.Fc show dose-dependent binding to its own antigen. The mean values for each group are derived from experiments done in triplicates and the s.d. is represented by error bars. (TIF) [file pone.0140691.s002.tif]

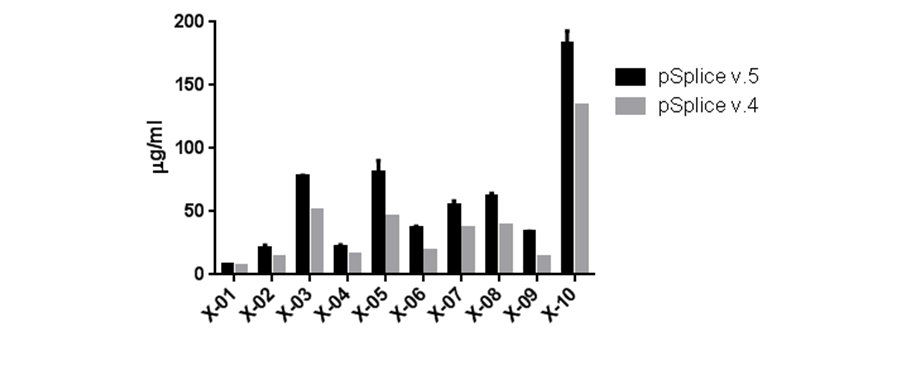

Supplement: S3 Fig — The mean values for each group are derived from experiments done in triplicates and the s.d. is represented by error bars. (TIF) [file pone.0140691.s003.tif]

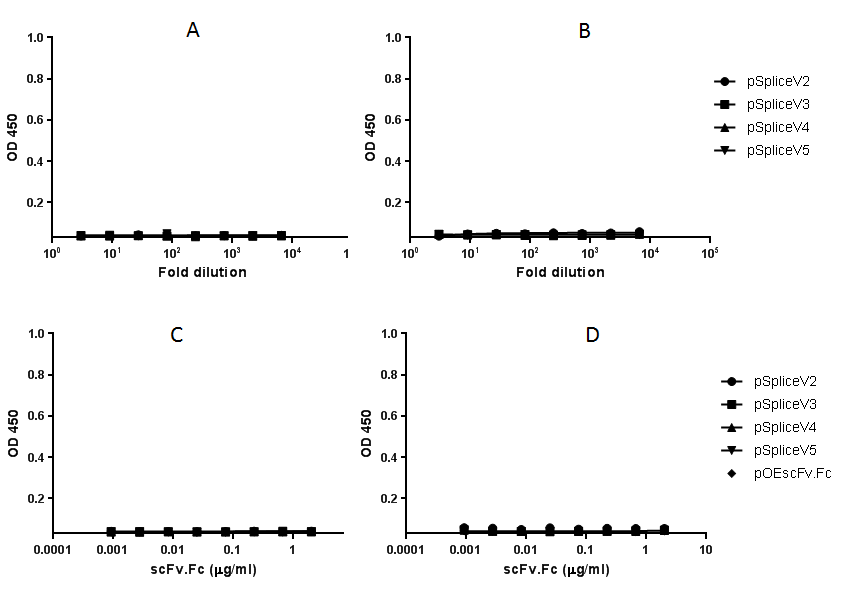

Supplement: S4 Fig — The mean values for each group are derived from experiments done in triplicates and the s.d. is represented by error bars. (TIF) [file pone.0140691.s004.tif]
